# Supplementary material for: Individual dynamic prediction of clinical endpoint from large dimensional longitudinal biomarker history: a landmark approach
Source: BMC Med Res Methodol. 2022 Jul 11;22:188. doi: 10.1186/s12874-022-01660-3 (PMC9275051; doi:10.1186/s12874-022-01660-3)
Supplement: Supplementary file 1 — Additional file 1 Additional information and results from simulations and applications. [file 12874_2022_1660_MOESM1_ESM.pdf]

Additional file

Anthony Devaux, Robin Genuer, Karine Pérès and Cécile Proust-Lima

May 27, 2022

1 Prediction methods for survival data

We consider the prediction methods using multiple settings, in particular the choice of the hyperparameters and/or the variable select, resulting to 11 sub-methods in total. In the following, we describe the difference between those sub-methods.

1.1 Cox models

Two cox models are estimated using either all predictors (*Cox-AllVar*) or some predictors (*Cox-SelectVar*) selected using a stepwise backward procedure based on the AIC statistic.

1.2 Penalized-Cox models

Penalized-cox models require the tuning of 2 parameters: the norm mixing parameter α and the penalty λ. The penalty λ is chosen by minimizing the partial likelihood deviance for a given α using an internal 10-folds cross-validation. We define 3 sub-models according to norm mixing parameter: lasso penalty with $\alpha = 1$ (*Penal-Cox-Lasso*), Ridge penalty with $\alpha = 0$ (*Penal-Cox-Ridge*) or elastic-net penalty with $\alpha \in [0, 1]$ (*Penal-Cox-Elastic*). For the elastic-net penalty, multiple α are evaluated according to a grid from 0 to 1 with a 0.1 step. As a final *Penal-Cox-Elastic* model, we retain the model with the lower partial likelihood deviance over all α from the grid.

1.3 Deviance residuals-based sparse-Partial Least Square

Deviance residuals-based sparse-Partial Least Square models require the tuning of 2 parameters: the number of components C and the sparsity controled by the lasso penalty parameter for each component η. The number of components C are chosen by maximizing the $iAUCsurvROC$ criteria (from the `plsRcox` R package) for a given η using an internal 5-folds cross-validation. We define 3 sub-models according to the lasso penalty parameter: no sparsity with $\eta = 0$ (*sPLS-NoSparse*), maximum sparsity with $\eta = 0.9$ (*sPLS-MaxSparse*) or mixing sparsity with $\eta \in [0, 0.9]$ (*sPLS-Optimize*). For the mixing sparsity, multiple η are evaluated according to a grid from 0 to 0.9 with a 0.1 step. As a final *sPLS-Optimize* model, we retain the model with the higher $iAUCsurvROC$ over all η from the grid.

1.4 Random Survival Forests

Random survival forest methods require the tuning of 2 parameters: the number of predictors drawn at each node M and the minimal node size S. We defined 3 sub-methods according to these parameters. The *RSF-Default* method uses the default parameters with M equals to the square root of the number of predictors and $S = 15$. In the *RSF-Optimize* and the *RSF-SelectVar* methods, the parameters are tuned according to a grid of values, M from 5 to the maximum of predictors with a 5 step and $S \in \{3, 15\}$. The best parameters are chosen by minimizing the out-of-bag error based on the $1 - C$, where C is Harrell's concordance index. For the *RSF-SelectVar*, a final random survival forest is computed using the predictors with $VIMP > 0.005$.

2 Simulations

2.1 Model formulation for time-dependent markers

For all scenarios described in the main manuscript, the 17 time-dependent markers were generated up to $t_{LM} = 4$ according to a linear mixed model defined as:

Marker 1

$$Y_{i1}(t_{ij}) = \beta_0 + \beta_1 * t_{ij} + \beta_2 * t_{ij}^2 + b_{i0} + b_{i1} * t_{ij} + b_{i2} * t_{ij}^2 + \epsilon_{ij} \tag{1}$$

where the fixed coefficients $\beta_0 = 1.5$, $\beta_1 = 2.0$, $\beta_2 = -1.2$, the random effects $b_i = (b_{i0}, b_{i1}, b_{i2})^\top \sim \mathcal{N}(0, D)$ with $D = \begin{pmatrix} 1.5^2 & 0 & 0 \\ 0 & 0.8^2 & 0 \\ 0 & 0 & 0.5^2 \end{pmatrix}$ and the measurement error $\epsilon_{ij} \sim \mathcal{N}(0, \sigma^2)$ and $\sigma = 1.1$.

Marker 2

$$Y_{i2}(t_{ij}) = \beta_0 + \beta_1 * \log(t_{ij} + 0.1) + b_{i0} + b_{i1} * \log(t_{ij} + 0.1) + \epsilon_{ij} \tag{2}$$

where the fixed coefficients $\beta_0 = 5.5$, $\beta_1 = -1.5$, the random effects $b_i = (b_{i0}, b_{i1})^\top \sim \mathcal{N}(0, D)$ with $D = \begin{pmatrix} 1.4^2 & 0 \\ 0 & 0.6^2 \end{pmatrix}$ and the measurement error $\epsilon_{ij} \sim \mathcal{N}(0, \sigma^2)$ and $\sigma = 0.9$.

Marker 3

$$Y_{i3}(t_{ij}) = \beta_0 + \beta_1 * \sqrt{(t_{ij} + 0.1)} + b_{i0} + b_{i1} * \sqrt{(t_{ij} + 0.1)} + \epsilon_{ij} \tag{3}$$

where the fixed coefficients $\beta_0 = 2.5$, $\beta_1 = 1.8$, the random effects $b_i = (b_{i0}, b_{i1})^\top \sim \mathcal{N}(0, D)$ with $D = \begin{pmatrix} 0.7^2 & 0 \\ 0 & 0.7^2 \end{pmatrix}$ and the measurement error $\epsilon_{ij} \sim \mathcal{N}(0, \sigma^2)$ and $\sigma = 1.1$.

Marker 4

$$Y_{i4}(t_{ij}) = \beta_0 + \beta_1 * t_{ij} + b_{i0} + b_{i1} * t_{ij} + \epsilon_{ij} \tag{4}$$

where the fixed coefficients $\beta_0 = 3.0$, $\beta_1 = 1.2$, the random effects $b_i = (b_{i0}, b_{i1})^\top \sim \mathcal{N}(0, D)$ with $D = \begin{pmatrix} 0.7^2 & 0 \\ 0 & 0.5^2 \end{pmatrix}$ and the measurement error $\epsilon_{ij} \sim \mathcal{N}(0, \sigma^2)$ and $\sigma = 1.3$.

Marker 5

$$Y_{i5}(t_{ij}) = \beta_0 + \beta_1 * t_{ij}^2 + b_{i0} + b_{i1} * t_{ij}^2 + \epsilon_{ij} \tag{5}$$

where the fixed coefficients $\beta_0 = 0.0$, $\beta_1 = 0.7$, the random effects $b_i = (b_{i0}, b_{i1})^\top \sim \mathcal{N}(0, D)$ with $D = \begin{pmatrix} 0.3^2 & 0 \\ 0 & 0.2^2 \end{pmatrix}$ and the measurement error $\epsilon_{ij} \sim \mathcal{N}(0, \sigma^2)$ and $\sigma = 0.9$.

Marker 6

$$Y_{i6}(t_{ij}) = \beta_0 + \beta_1 * t_{ij} + \beta_2 * t_{ij}^2 + b_{i0} + b_{i1} * t_{ij} + b_{i2} * t_{ij}^2 + \epsilon_{ij} \tag{6}$$

where the fixed coefficients $\beta_0 = 3.5$, $\beta_1 = -1.2$, $\beta_2 = 0.8$, the random effects $b_i = (b_{i0}, b_{i1}, b_{i2})^\top \sim \mathcal{N}(0, D)$ with $D = \begin{pmatrix} 1.5^2 & 0 & 0 \\ 0 & 0.7^2 & 0 \\ 0 & 0 & 0.5^2 \end{pmatrix}$ and the measurement error $\epsilon_{ij} \sim \mathcal{N}(0, \sigma^2)$ and $\sigma = 1.1$.

Marker 7

$$Y_{i7}(t_{ij}) = \beta_0 + \beta_1 * t_{ij} + b_{i0} + b_{i1} * t_{ij} + \epsilon_{ij} \tag{7}$$

where the fixed coefficients $\beta_0 = 1.1$, $\beta_1 = 0.8$, the random effects $b_i = (b_{i0}, b_{i1})^\top \sim \mathcal{N}(0, D)$ with $D = \begin{pmatrix} 0.3^2 & 0 \\ 0 & 0.5^2 \end{pmatrix}$ and the measurement error $\epsilon_{ij} \sim \mathcal{N}(0, \sigma^2)$ and $\sigma = 0.5$.

Marker 8

$$Y_{i8}(t_{ij}) = \beta_0 + \beta_1 * \exp(t_{ij}) + b_{i0} + b_{i1} * \exp(t_{ij}) + \epsilon_{ij} \tag{8}$$

where the fixed coefficients $\beta_0 = 1.1$, $\beta_1 = -0.2$, the random effects $b_i = (b_{i0}, b_{i1})^\top \sim \mathcal{N}(0, D)$ with $D = \begin{pmatrix} 0.3^2 & 0 \\ 0 & 0.1^2 \end{pmatrix}$ and the measurement error $\epsilon_{ij} \sim \mathcal{N}(0, \sigma^2)$ and $\sigma = 0.5$.

Marker 9

$$Y_{i9}(t_{ij}) = \beta_0 + \beta_1 * \log(t_{ij} + 0.1) + \beta_2 * t_{ij}^2 + b_{i0} + b_{i1} * \log(t_{ij} + 0.1) + b_{i2} * t_{ij}^2 + \epsilon_{ij} \tag{9}$$

where the fixed coefficients $\beta_0 = 6.5$, $\beta_1 = 4.5$, $\beta_2 = -1.0$, the random effects $b_i = (b_{i0}, b_{i1}, b_{i2})^\top \sim \mathcal{N}(0, D)$ with $D = \begin{pmatrix} 1.2^2 & 0 & 0 \\ 0 & 2.5^2 & 0 \\ 0 & 0 & 0.3^2 \end{pmatrix}$ and the measurement error $\epsilon_{ij} \sim \mathcal{N}(0, \sigma^2)$ and $\sigma = 0.9$.

Marker 10

$$Y_{i10}(t_{ij}) = \beta_0 + \beta_1 * t_{ij} + \beta_2 * t_{ij}^2 + b_{i0} + b_{i1} * t_{ij} + b_{i2} * t_{ij}^2 + \epsilon_{ij} \tag{10}$$

where the fixed coefficients $\beta_0 = 4.1$, $\beta_1 = -2.0$, $\beta_2 = 0.9$, the random effects $b_i = (b_{i0}, b_{i1}, b_{i2})^\top \sim \mathcal{N}(0, D)$ with $D = \begin{pmatrix} 1.5^2 & 0 & 0 \\ 0 & 1.1^2 & 0 \\ 0 & 0 & 0.4^2 \end{pmatrix}$ and the measurement error $\epsilon_{ij} \sim \mathcal{N}(0, \sigma^2)$ and $\sigma = 1.1$.

Marker 11

$$Y_{i11}(t_{ij}) = \beta_0 + \beta_1 * t_{ij} + \beta_2 * t_{ij}^2 + b_{i0} + b_{i1} * t_{ij} + b_{i2} * t_{ij}^2 + \epsilon_{ij} \tag{11}$$

where the fixed coefficients $\beta_0 = 9.4$, $\beta_1 = -1.2$, $\beta_2 = -0.7$, the random effects $b_i = (b_{i0}, b_{i1}, b_{i2})^\top \sim \mathcal{N}(0, D)$ with $D = \begin{pmatrix} 0.9^2 & 0 & 0 \\ 0 & 0.7^2 & 0 \\ 0 & 0 & 0.8^2 \end{pmatrix}$ and the measurement error $\epsilon_{ij} \sim \mathcal{N}(0, \sigma^2)$ and $\sigma = 1.1$.

Marker 12

$$Y_{i12}(t_{ij}) = \beta_0 + \beta_1 * \sqrt{(t_{ij} + 0.1)} + b_{i0} + b_{i1} * \sqrt{(t_{ij} + 0.1)} + \epsilon_{ij} \tag{12}$$

where the fixed coefficients $\beta_0 = 5.2$, $\beta_1 = 4.7$, the random effects $b_i = (b_{i0}, b_{i1})^\top \sim \mathcal{N}(0, D)$ with $D = \begin{pmatrix} 0.9^2 & 0 \\ 0 & 1.1^2 \end{pmatrix}$ and the measurement error $\epsilon_{ij} \sim \mathcal{N}(0, \sigma^2)$ and $\sigma = 0.9$.

Marker 13

$$Y_{i13}(t_{ij}) = \beta_0 + \beta_1 * \sqrt{(t_{ij}+0.1)} + b_{i0} + b_{i1} * \sqrt{(t_{ij}+0.1)} + \epsilon_{ij} \quad (13)$$

where the fixed coefficients $\beta_0 = 8.2$, $\beta_1 = -3.2$, the random effects $b_i = (b_{i0}, b_{i1})^\top \sim \mathcal{N}(0, D)$ with $D = \begin{pmatrix} 1.3^2 & 0 \\ 0 & 1.6^2 \end{pmatrix}$ and the measurement error $\epsilon_{ij} \sim \mathcal{N}(0, \sigma^2)$ and $\sigma = 1.3$.

Marker 14

$$Y_{i14}(t_{ij}) = \beta_0 + \beta_1 * t_{ij} + \beta_2 * t_{ij}^3 + b_{i0} + b_{i1} * t_{ij} + b_{i2} * t_{ij}^3 + \epsilon_{ij} \quad (14)$$

where the fixed coefficients $\beta_0 = 3.6$, $\beta_1 = -0.9$, $\beta_2 = 0.4$, the random effects $b_i = (b_{i0}, b_{i1}, b_{i2})^\top \sim \mathcal{N}(0, D)$ with $D = \begin{pmatrix} 1.5^2 & 0 & 0 \\ 0 & 0.5^2 & 0 \\ 0 & 0 & 0.1^2 \end{pmatrix}$ and the measurement error $\epsilon_{ij} \sim \mathcal{N}(0, \sigma^2)$ and $\sigma = 1.1$.

Marker 15

$$Y_{i15}(t_{ij}) = \beta_0 + \beta_1 * t_{ij} + \beta_2 * t_{ij}^3 + b_{i0} + b_{i1} * t_{ij} + b_{i2} * t_{ij}^3 + \epsilon_{ij} \quad (15)$$

where the fixed coefficients $\beta_0 = 8.6$, $\beta_1 = 4.9$, $\beta_2 = -0.4$, the random effects $b_i = (b_{i0}, b_{i1}, b_{i2})^\top \sim \mathcal{N}(0, D)$ with $D = \begin{pmatrix} 1.5^2 & 0 & 0 \\ 0 & 0.7^2 & 0 \\ 0 & 0 & 0.2^2 \end{pmatrix}$ and the measurement error $\epsilon_{ij} \sim \mathcal{N}(0, \sigma^2)$ and $\sigma = 1.1$.

Marker 16

$$Y_{i16}(t_{ij}) = \beta_0 + \beta_1 * \exp(t_{ij}) + b_{i0} + b_{i1} * \exp(t_{ij}) + \epsilon_{ij} \quad (16)$$

where the fixed coefficients $\beta_0 = 4.1$, $\beta_1 = -0.2$, the random effects $b_i = (b_{i0}, b_{i1})^\top \sim \mathcal{N}(0, D)$ with $D = \begin{pmatrix} 0.6^2 & 0 \\ 0 & 0.2^2 \end{pmatrix}$ and the measurement error $\epsilon_{ij} \sim \mathcal{N}(0, \sigma^2)$ and $\sigma = 0.5$.

Marker 17

$$Y_{i17}(t_{ij}) = \beta_0 + \beta_1 * t_{ij}^3 + b_{i0} + b_{i1} * t_{ij}^3 + \epsilon_{ij} \tag{17}$$

where the fixed coefficients $\beta_0 = 3.2$, $\beta_1 = 0.3$, the random effects $b_i = (b_{i0}, b_{i1})^\top \sim \mathcal{N}(0, D)$ with $D = \begin{pmatrix} 0.6^2 & 0 \\ 0 & 0.2^2 \end{pmatrix}$ and the measurement error $\epsilon_{ij} \sim \mathcal{N}(0, \sigma^2)$ and $\sigma = 1.0$.

We display an example of individual trajectories for all markers in Fig. S1. In the following for the sake of simplicity, we denote the summaries Y_k^{pred}, Y_k^{slope}, Y_k^{hist} for respectively the current level, current slope and history level on landmark time for the marker k.

2.2 Model formulation for time-to-event

We generated the hazard function λ_i according to a proportional hazard model defined as:

$$\lambda_i(t) = \lambda_0(b, c, t) \exp(\mathcal{P}_i) \tag{18}$$

With $\lambda_0(b, c, t) = cb^c t^{c-1}$ the baseline hazard function from a Weibull distribution with parameters b and c, and $\mathcal{P}_i$ the linear predictor. Using the standard uniform distribution $u \sim \mathcal{U}(0, 1)$ [1], we generated time-to-event T_i defined as:

$$T_i = \frac{1}{b} * \left(-\frac{\log u}{\exp(\mathcal{P}_i)} \right)^{1/c} \tag{19}$$

In the following, we detail the parameters used to build the hazard function from equation 18 in each of the scenarios. A recap of the summaries used in each scenario is available in the table S1.

Scenario 1: few summaries with linear association

$$\mathcal{P}_i = \gamma_1 * Y_{1i}^{pred} + \gamma_2 * Y_{3i}^{hist} + \gamma_1 * Y_{8i}^{pred} + \gamma_2 * Y_{15i}^{pred} \tag{20}$$

With $\gamma_1 = -0.5$ and $\gamma_2 = 0.5$ the coefficients associated to the summaries and $b = 0.3$ and $c = 6.5$ to build the base hazard function λ_0. Results from this scenario are given in Fig. S2A.

Scenario 2: few summaries with linear association with interaction

$$\begin{aligned}\mathcal{P}_i &= \gamma_1 * Y_{1i}^{pred} + \gamma_2 * Y_{3i}^{hist} + \gamma_1 * Y_{8i}^{pred} + \gamma_2 * Y_{15i}^{pred} + \gamma_1 * Y_{1i}^{pred} * Y_{3i}^{hist} \\ &+ \gamma_2 * Y_{8i}^{pred} * Y_{15i}^{pred} + \gamma_1 * Y_{1i}^{pred} * Y_{8i}^{pred} + \gamma_2 * Y_{3i}^{hist} * Y_{15i}^{pred}\end{aligned} \tag{21}$$

With $\gamma_1 = -0.5$ and $\gamma_2 = 0.5$ denote the coefficients associated to the summaries and $b = 0.3$ and $c = 6.5$ to build hazard function λ_0. Results from this scenario are given in Fig. S2B.

Scenario 3: few summaries with non-linear association

$$\mathcal{P}_i = \gamma_1 * (Y_{1i}^{pred})^2 + \gamma_1 * (Y_{1i}^{slope})^2 + \gamma_1 * (Y_{4i}^{slope})^2 + \gamma_1 * (Y_{10i}^{pred})^2 \tag{22}$$

With $\gamma_1 = 0.5$ denotes the coefficients associated to the summaries and $b = 0.25$ and $c = 6.5$ to build hazard function λ_0. Results from this scenario are given in Fig. S2C.

Scenario 4: many summaries with linear association

$$\begin{aligned}\mathcal{P}_i &= \gamma_1 * Y_{1i}^{pred} + \gamma_2 * Y_{1i}^{slope} + \gamma_1 * Y_{3i}^{pred} + \gamma_2 * Y_{3i}^{hist} + \gamma_1 * Y_{4i}^{pred} + \gamma_2 * Y_{4i}^{slope} \\ &+ \gamma_1 * Y_{5i}^{pred} + \gamma_2 * Y_{5i}^{slope} + \gamma_1 * Y_{5i}^{hist} + \gamma_2 * Y_{10i}^{pred} + \gamma_1 * Y_{10i}^{hist} + \gamma_2 * Y_{13i}^{pred} \\ &+ \gamma_1 * Y_{13i}^{slope} + \gamma_2 * Y_{15i}^{pred} + \gamma_1 * Y_{15i}^{slope} + \gamma_2 * Y_{15i}^{hist} + \gamma_1 * Y_{17i}^{pred} + \gamma_2 * Y_{17i}^{slope}\end{aligned} \tag{23}$$

With $\gamma_1 = -0.5$ and $\gamma_2 = 0.5$ denote the coefficients associated to the summaries and $b = 0.3$ and $c = 6.5$ to build hazard function λ_0. Results from this scenario are given in the main manuscript.

Scenario 5: many summaries with non-linear association

$$\begin{aligned}\mathcal{P}_i &= \gamma_1 * (Y_{1i}^{pred})^2 + \gamma_1 * (Y_{1i}^{slope})^2 + \gamma_2 * (Y_{2i}^{slope})^2 + \gamma_2 * (Y_{3i}^{hist})^2 + \gamma_1 * (Y_{4i}^{pred})^2 \\ &+ \gamma_2 * (Y_{4i}^{slope})^2 + \gamma_1 * (Y_{10i}^{pred})^2 + \gamma_2 * (Y_{11i}^{hist})^2 + \gamma_1 * (Y_{12i}^{slope})^2 + \gamma_2 * (Y_{13i}^{pred})^2 \\ &+ \gamma_1 * (Y_{14i}^{slope})^2 + \gamma_2 * \mathbb{1}(Y_{15i}^{pred} > \tilde{Y}_{15}^{pred}) + \gamma_1 * \mathbb{1}(Y_{16i}^{hist} > \tilde{Y}_{16}^{hist}) \\ &+ \gamma_2 * \mathbb{1}(Y_{17i}^{pred} > \tilde{Y}_{17}^{pred}) + \gamma_1 * \mathbb{1}(Y_{5i}^{slope} > \tilde{Y}_{5}^{slope}) + \gamma_2 * \mathbb{1}(Y_{6i}^{hist} > \tilde{Y}_{6}^{hist}) \\ &+ \gamma_1 * \mathbb{1}(Y_{9i}^{slope} > \tilde{Y}_{9}^{slope}) + \gamma_2 * \mathbb{1}(Y_{9i}^{hist} > \tilde{Y}_{9}^{hist})\end{aligned} \tag{24}$$

With $\gamma_1 = -0.5$ and $\gamma_2 = 0.5$ denote the coefficients associated to the summaries and $b = 0.28$ and $c = 5.5$ to build hazard function λ_0. $\tilde{Y}_k$ represents the median for marker k. Results from this scenario are given in the main manuscript.

3 Applications

3.1 Prediction of death in primary billiary cholangitis

To estimate the probability of death on primary biliary cholangitis patients, we used 7 continuous time-dependent markers measuring bilirubin, cholesterol, albumin, alkaline, SGOT, platelets and prothrombin and 4 binary time-dependent markers measuring the presence of ascites, hepatomegaly, spiders and edema. Except albumin, all continuous variables were normalized using splines to follow a gaussian distribution [2].

Except ascites and edema, we modeled the variables using generalized mixed model defined as:

$$g(E(Y_{ij}|b_i)) = \beta_0 + \sum_{l=1}^{L} \beta_l * f_l(t, L) + b_{i0} + \sum_{l=1}^{L} b_{il} * f_l(t, L) \tag{25}$$

With $g(.)$ the link function taking into account the nature of the marker, β_0 and β_l the fixed coefficients and the random effects $b_{il} = (b_{i0}, b_{il})^\top \sim \mathcal{N}(0, D)$ with D a covariance matrix. $f(t, L)$ denotes the natural splines function with L knots.

To model continuous markers, we use $g(.)$ as the identity function, D an unstructured covariance matrix and $L = 3$ for the natural splines function with two internal knots placed at $t = 0.5$ and $t = 2.0$ and boundary knots at $t = 0$ and $t = 4$. To model binary markers, we use $g(.)$ as the logit function, D a diagonal independent covariance matrix and $L = 2$ for the natural splines function with a single internal knot placed at $t = 1.0$ and boundary knots at $t = 0$ and $t = 4$.

Finally, to avoid convergence issues, ascites and edema are defined as:

$$g(E(Y_{ij}|b_i)) = \beta_0 + \beta_1 * t_{ij} + b_{i0} + b_{i1} * t_{ij} \tag{26}$$

With $g(.)$ the logit function, β_0 and β_l the fixed coefficients and the random effects $b_{il} = (b_{i0}, b_{i1})^\top \sim \mathcal{N}(0, D)$ with D an unstructured covariance matrix.

3.2 Prediction of 5-years death at 80 and 85 years old

3.2.1 Model specification

To estimate the probability of death at 80 and 85 years old, we used 6 continuous time-dependent markers (measuring depression, executive functioning, cognition, speed on fluency, dependency and polymedication) and 3 binary time-dependent markers (measuring the presence of incontinence, dyspnea and living alone). Except executive functioning and speed on fluency, all continuous variables were normalized using splines to follow a gaussian distribution [2]. We modeled the variables from 5 years prior the landmark time using generalized mixed model defined as:

$$g(E(Y_{ij}|b_i)) = \beta_0 + \sum_{l=1}^{L} \beta_l * f_l(t, L) + b_{i0} + \sum_{l=1}^{L} b_{il} * f_l(t, L) \tag{27}$$

With $g(.)$ the link function taking into account the nature of the marker, β_0 and β_l the fixed coefficients and the random effects $b_{il} = (b_{i0}, b_{il})^\top \sim \mathcal{N}(0, D)$ with D a covariance matrix. $f(t, L)$ denotes the natural splines function with L knots.

To model continuous markers, we use $g(.)$ as the identity function, D an unstructured covariance matrix and $L = 3$ for the natural splines function with two internal knots placed at $t = 1.7$ and $t = 3.4$ and boundary knots at $t = 0$ and $t = 5$.

To avoid convergence issues, we model binary markers as:

$$g(E(Y_{ij}|b_i)) = \beta_0 + \beta_1 * t_{ij} + b_{i0} + b_{i1} * t_{ij} \tag{28}$$

With $g(.)$ the logit function, β_0 and β_l the fixed coefficients and the random effects $b_{il} = (b_{i0}, b_{i1})^\top \sim \mathcal{N}(0, D)$ with D a diagonal independent covariance matrix.

3.2.2 Age-specific predictors of death using Cox model with Lasso penalty

Fig. S14 displays the predictors selected for Cox model with Lasso penalty. We can see that many time-dependent markers are associated with death, especially with $t_{LM} = 80$. In addition, among these markers, we found 3 predictors of death (measuring executive functioning, dependency and polymedication) at both landmark time 80 and 85

years old. Variables measuring dependency and polymedication are strongly predictive of death at $t_{LM} = 80$. Indeed, 3 summaries were selected by the model. For time-independent variables, sex, history of dementia and dependency are predictive of death at both landmark time 80 and 85 years old.

4 Software

All analysis were performed using R software version 3.6. We used `lcmm` [3] (for continuous markers) and `lme4` [4] (for binary markers) to compute generalized mixed models. Predictions are computed using `survival` [5] for Cox model, `glmnet` [6] for Cox model with penalty (with 2 hyperparameters: λ and α for respectively strength of the penalty and the type of penalty), `plsRcox` [7] for the Deviance residuals-based sparse-Partial Least Square (with 2 hyperparameters: η and *ncomp* for respectively sparsity parameter and the number of components) and `randomForestSRC` [8] for random survival forests (with 2 hyperparameters: *mtry* and *nodesize* for respectively the number of variables randomly selected as candidates for splitting a node and the forest average number of unique cases in a terminal node). R code detail and example can be found on https://github.com/anthonydevaux/hdlandmark.

References

[1] Bender, R., Augustin, T., Blettner, M.: Generating survival times to simulate Cox proportional hazards models. Statistics in Medicine **24**(11), 1713–1723 (2005). doi:10.1002/sim.2059

[2] Philipps, V., Amieva, H., Andrieu, S., Dufouil, C., Berr, C., Dartigues, J.-F., Jacqmin-Gadda, H., Proust-Lima, C.: Normalized Mini-Mental State Examination for Assessing Cognitive Change in Population-Based Brain Aging Studies. Neuroepidemiology **43**(1), 15–25 (2014). doi:10.1159/000365637

[3] Proust-Lima, C., Philipps, V., Liquet, B.: Estimation of Extended Mixed Models Using Latent Classes and Latent Processes: The R Package lcmm. Journal of Statistical Software **78**(2), 1–56 (2017). doi:10.18637/jss.v078.i02

[4] Bates, D., Mächler, M., Bolker, B., Walker, S.: Fitting Linear Mixed-Effects Models Using lme4. Journal of Statistical Software **67**(1), 1–48 (2015). doi:10.18637/jss.v067.i01

[5] Terry M. Therneau, Patricia M. Grambsch: Modeling Survival Data: Extending the Cox Model. Springer, New York (2000)

[6] Simon, N., Friedman, J., Hastie, T., Tibshirani, R.: Regularization Paths for Cox's Proportional Hazards Model via Coordinate Descent. Journal of Statistical Software **39**(5) (2011). doi:10.18637/jss.v039.i05

[7] Bastien, P., Bertrand, F., Meyer, N., Maumy-Bertrand, M.: Deviance residuals-based sparse PLS and sparse kernel PLS regression for censored data. Bioinformatics **31**(3), 397–404 (2015). doi:10.1093/bioinformatics/btu660

[8] Ishwaran, H., Kogalur, U.B., Blackstone, E.H., Lauer, M.S.: Random survival forests. The Annals of Applied Statistics **2**(3), 841–860 (2008). doi:10.1214/08-AOAS169

Figures

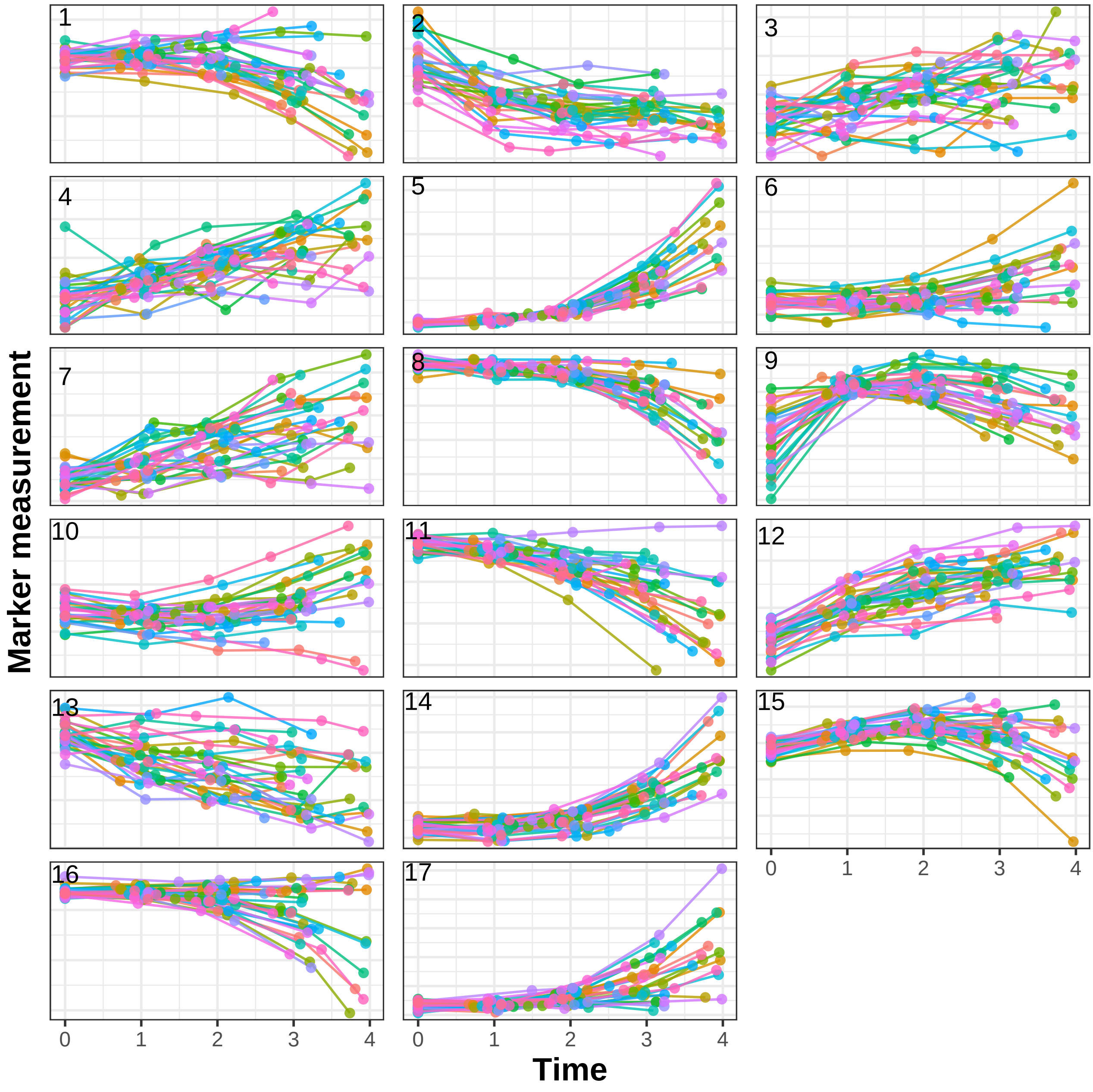

Figure S1: Illustration of 30 randomly selected individual trajectories chosen randomly for the 17 markers generated up to $t_{LM} = 4$ in the simulation study.

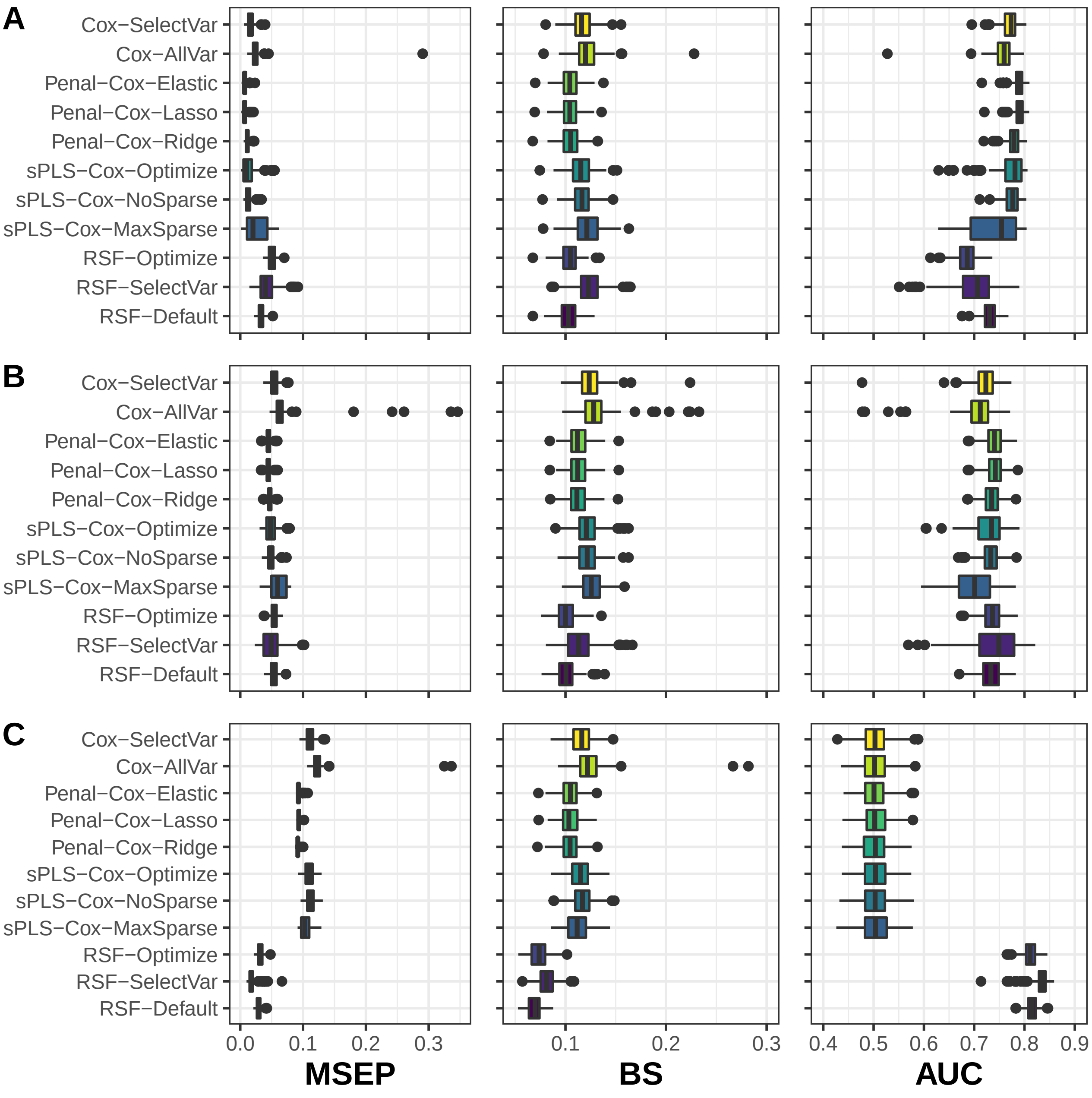

Figure S2: Scenario results with 4 summaries associated to the event with linear form (figure A), linear form with interaction (figure B) and non-linear form (figure C) over 250 replicates. Methods are assessed using at 3 years Mean Square Error of Prediction (MSEP), Brier Score (BS) and Area Under the ROC Curve (AUC).

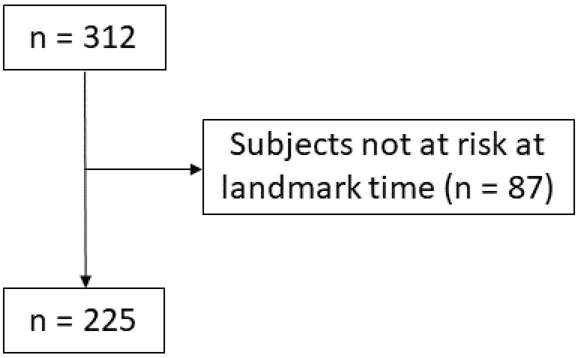

Figure S3: PBC data flowchart

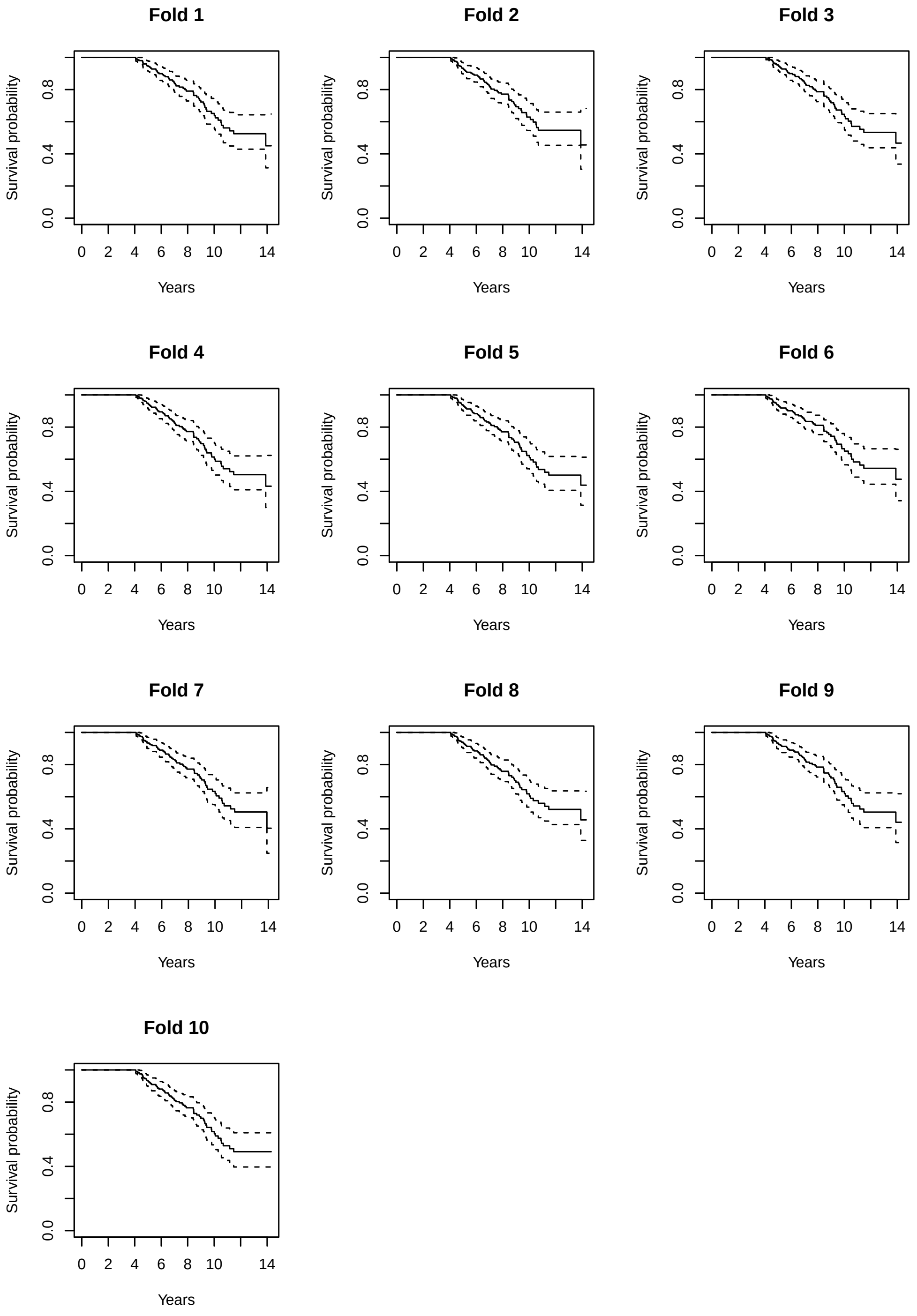

Figure S4: 3-year survival probability estimated by Kaplan-Meier in each of the 10 folds for PBC subjects still at risk at landmark time $t_{LM} = 4$.

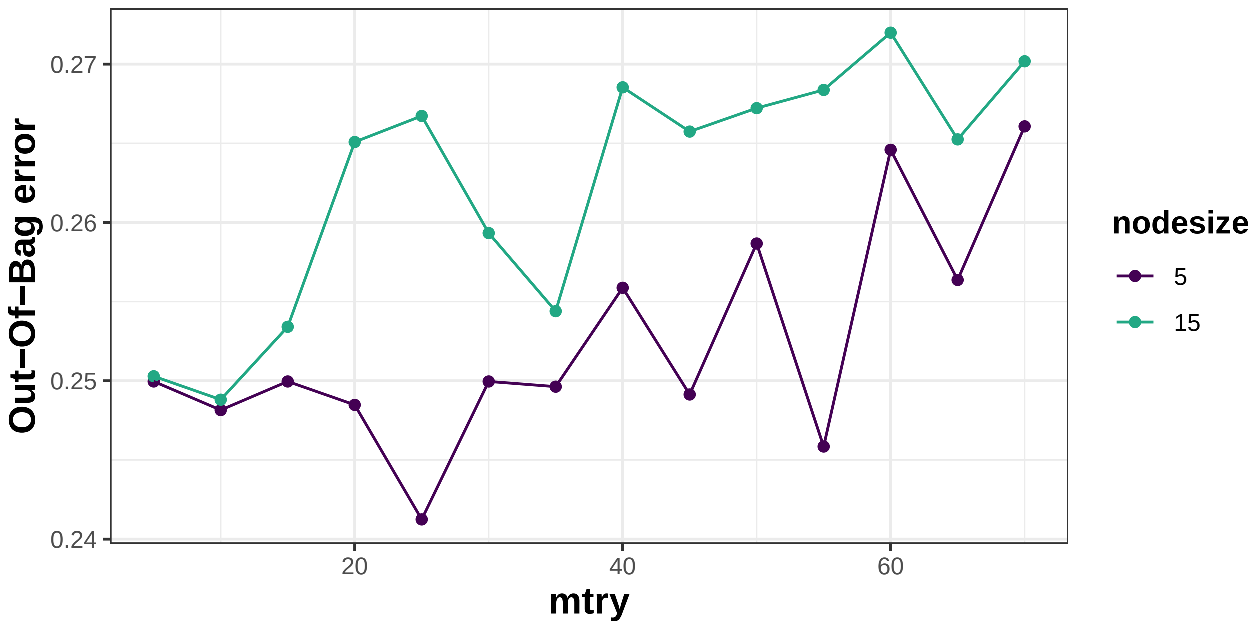

Figure S5: Random survival forest hyperparameters tuning on primary biliary cholangitis patients at landmark time $t_{LM} = 4$. The best hyperparameters ($mtry = 25$ and $nodesize = 5$) are chosen by minimizing the out-of-bag error.

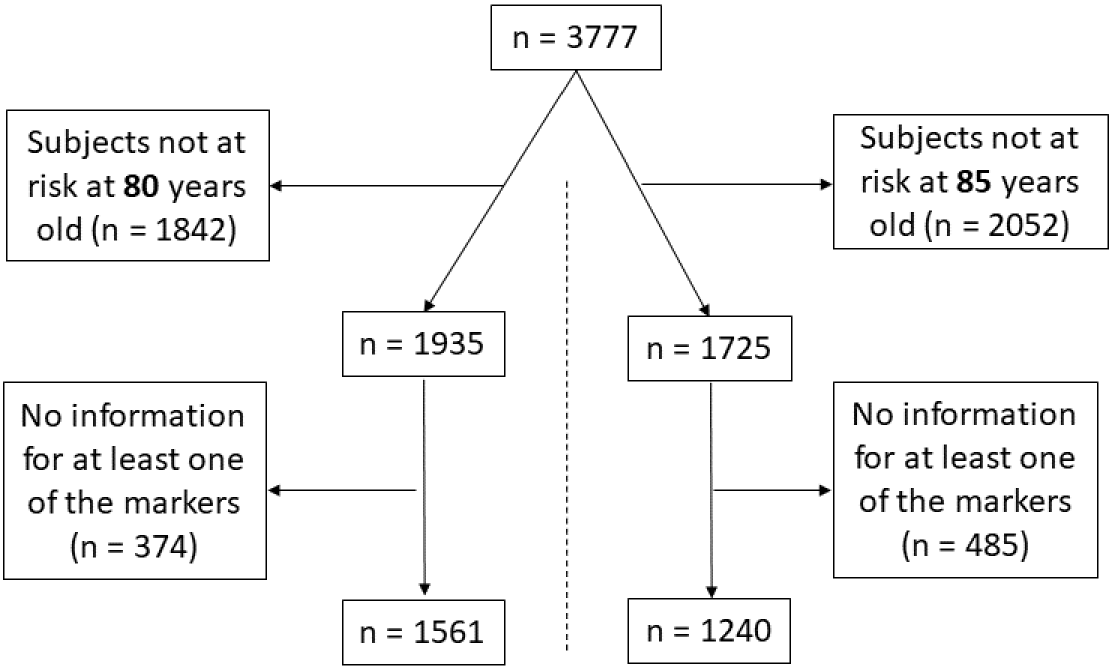

Figure S6: Flowchart for Paquid application with a landmark times at 80 (left) and 85 (right) years old.

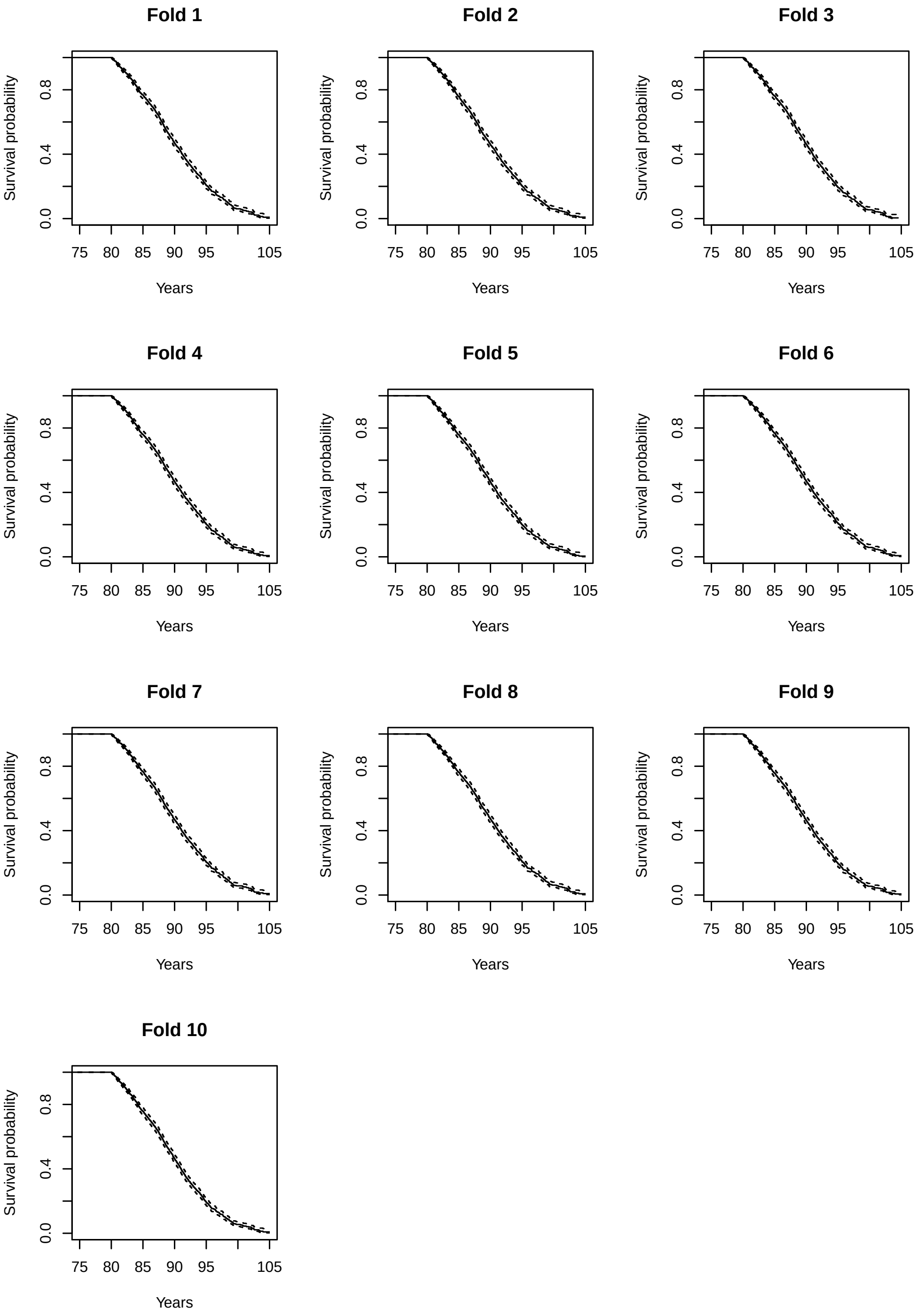

Figure S7: Survival probability estimated by Kaplan-Meier over the 10 folds for elderly people still at risk at landmark time $t_{LM} = 80$.

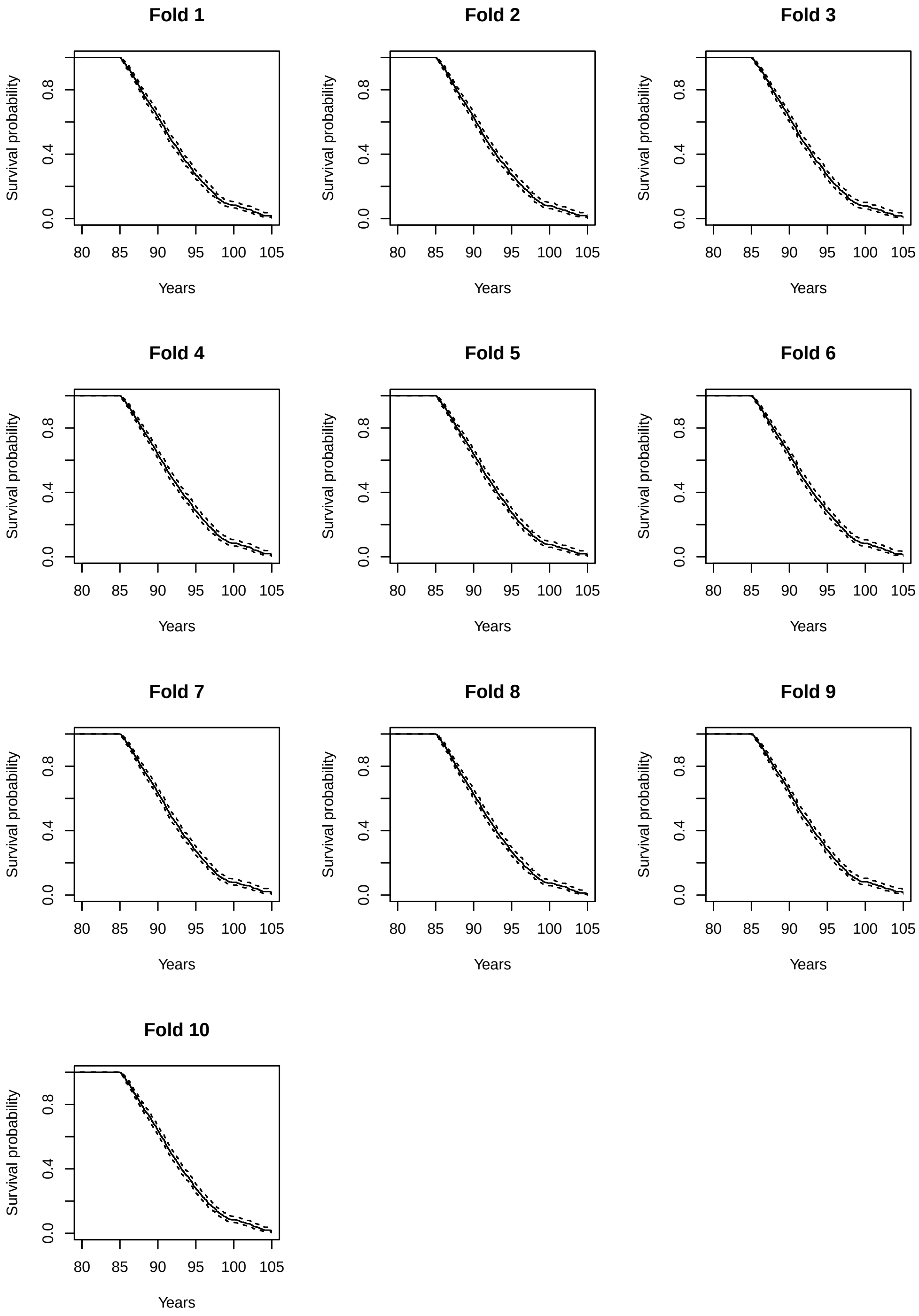

Figure S8: Survival probability estimated by Kaplan-Meier over the 10 folds for elderly people still at risk at landmark time $t_{LM} = 85$.

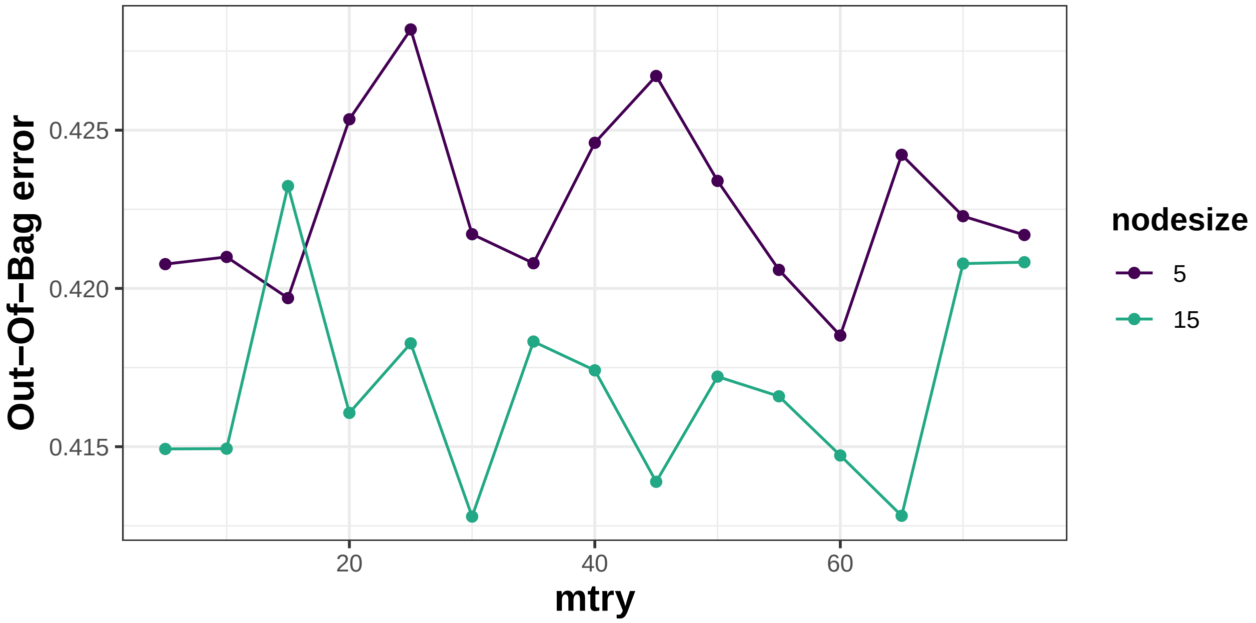

Figure S9: Random survival forest hyperparameters tuning in the paquid application at landmark time $t_{LM} = 80$. The best hyperparameters ($mtry = 30$ and $nodesize = 15$) are chosen by minimizing the out-of-bag error.

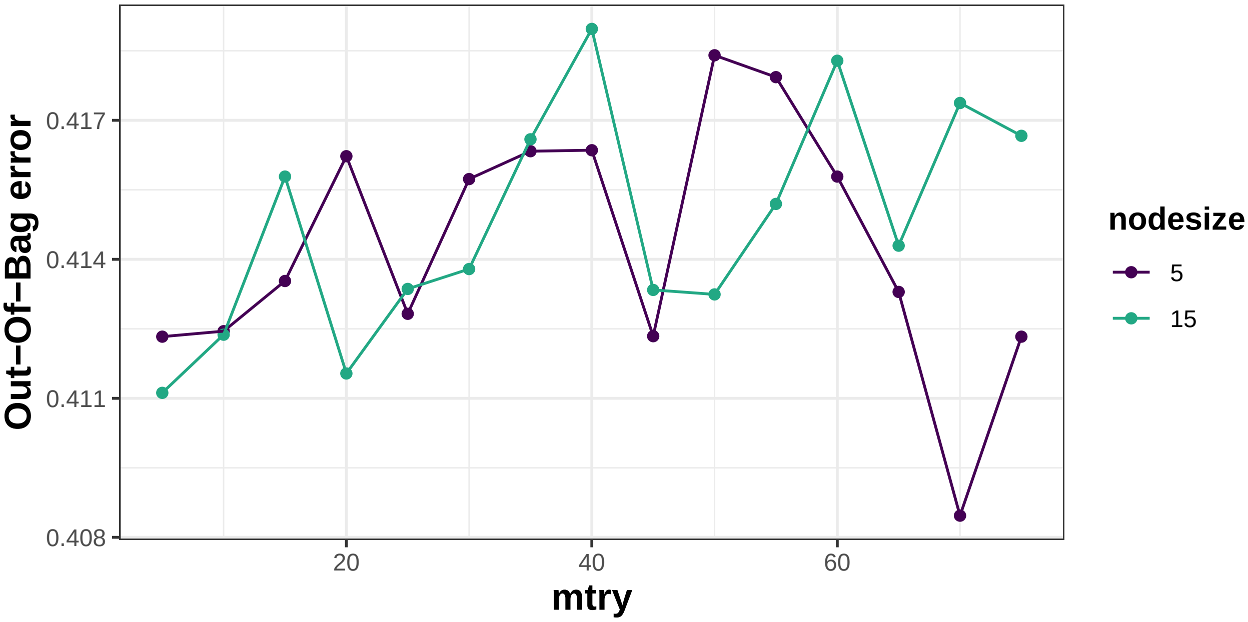

Figure S10: Random survival forest hyperparameters tuning in the paquid application at landmark time $t_{LM} = 85$. The best hyperparameters ($mtry = 70$ and $nodesize = 5$) are chosen by minimizing the out-of-bag error.

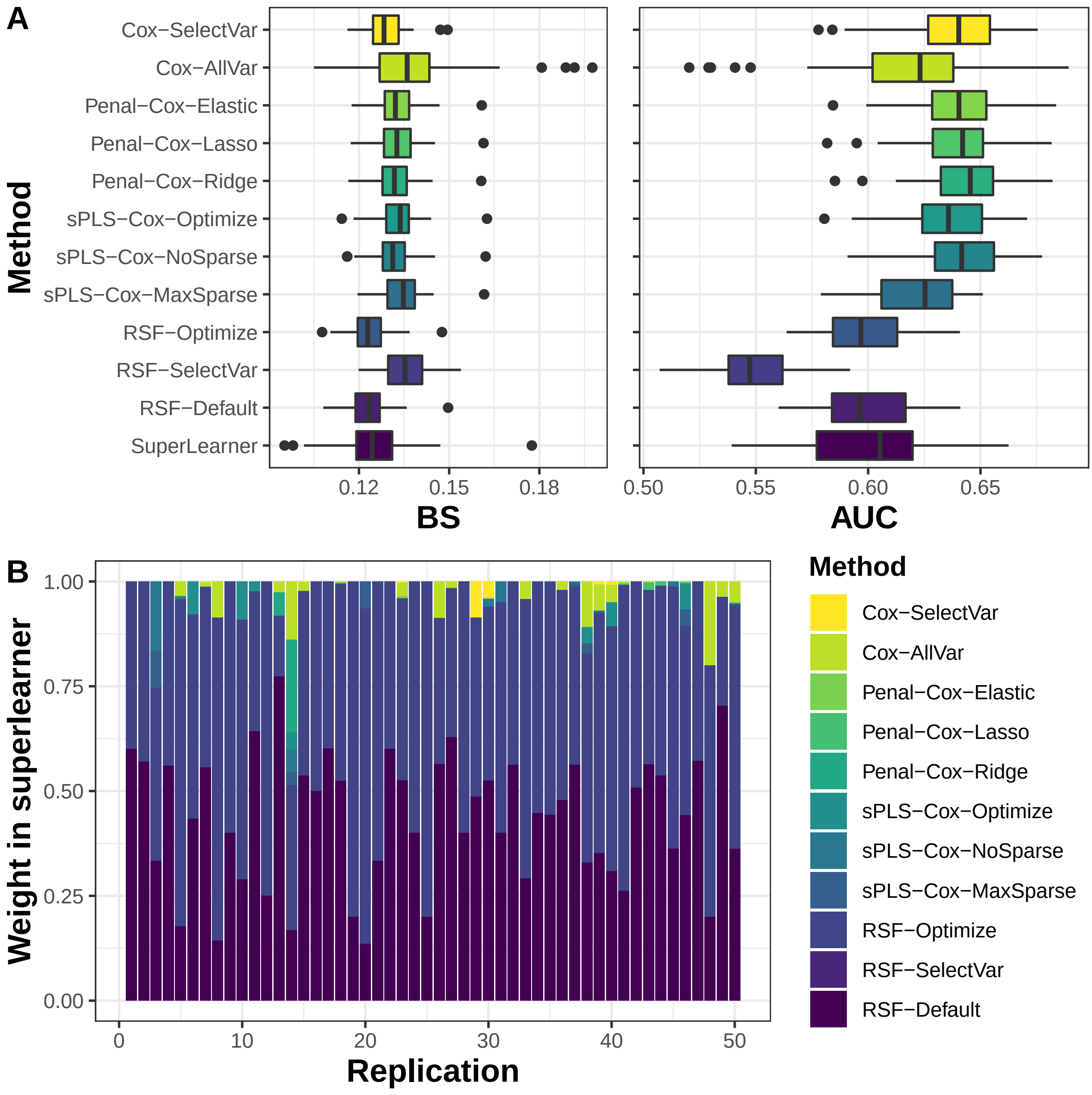

Figure S11: Predictive performances (figure A) and weights in superlearner (figure B) of 5-year survival prediction tool that uses information collected from the last 5 years before landmark time $t_{LM} = 80$ over 50 replicates. Methods are assessed using Brier Score (BS) and Area Under the ROC Curve (AUC).

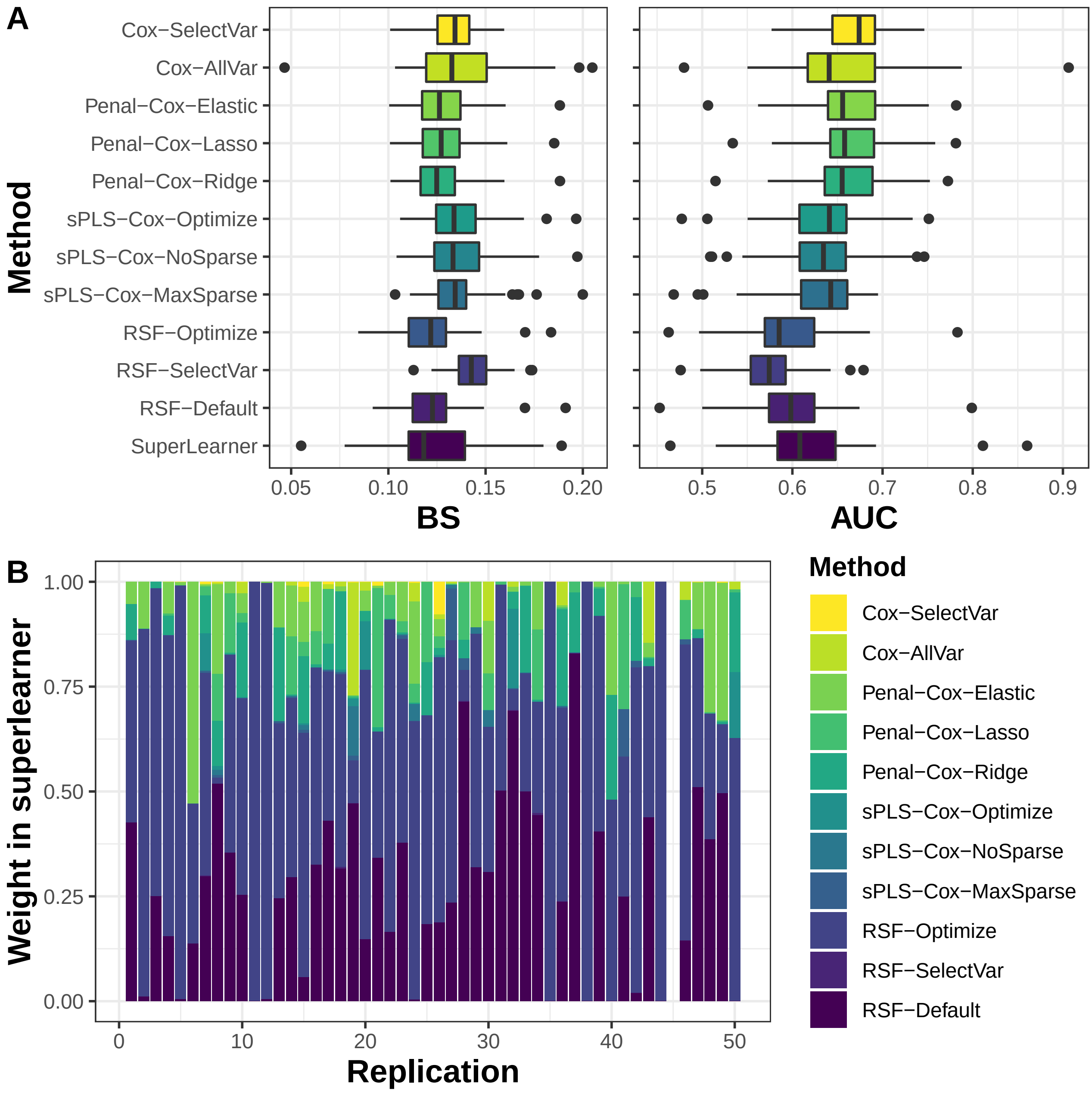

Figure S12: Predictive performances (figure A) and weights in superlearner (figure B) of 5-year survival prediction tools that use information collected from the last 5 years before landmark time $t_{LM} = 85$ over 50 replicates. Methods are assessed using Brier Score (BS) and Area Under the ROC Curve (AUC).

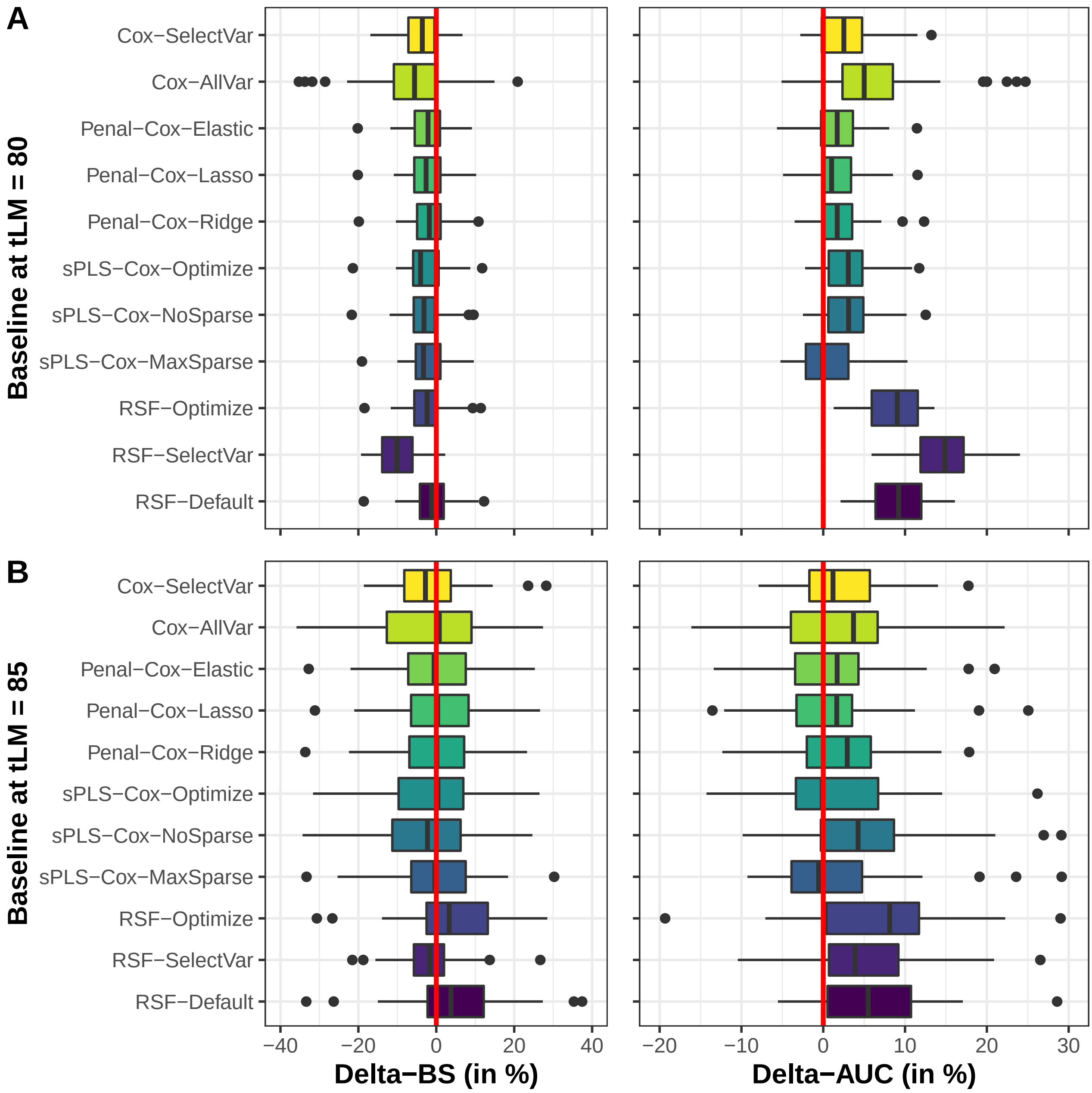

Figure S13: Predictive performances of 5-year survival prediction tools that use information collected at landmark time $t_{LM} = 80$ (figure A) and $t_{LM} = 85$ (figure B) over 50 replicates. Methods are assessed using Brier Score (BS) and Area Under the ROC Curve (AUC).

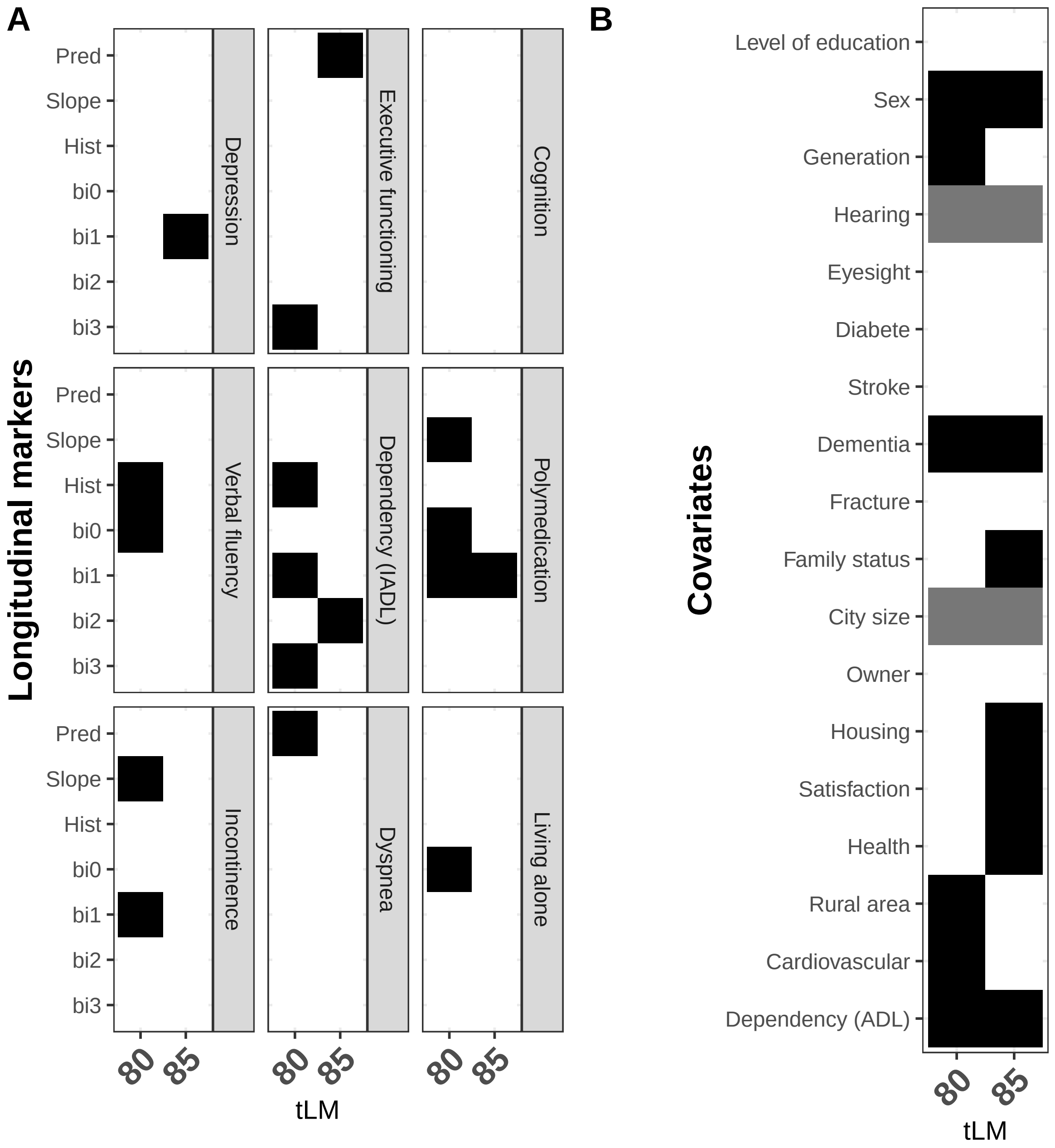

Figure S14: Variables associated with the event for Cox model with Lasso penalty. The heatmaps show which summaries (figure A) and covariates (figure B) have been selected in the model. The black color indicates that the variable has been selected, while the color grey indicates that at least one modality of the variable has been selected, otherwise white for no selection.

Tables

Table S1: Type of summaries used in scenario 1 (in red), scenario 2 (in blue), scenario3 (in green), scenario 4 (in yellow) or scenario 5 (in orange).

	Y_i^{pred}	Y_i^{slope}	Y_i^{hist}	b_{i0}	b_{i1}	b_{i2}
Marker 1	✔ (red) ✔ (blue) ✔ (green)	✔ (green) ✔ (yellow) ✔ (orange)	✔ (yellow) ✔ (orange)			
Marker 2		✔ (orange)				
Marker 3	✔ (yellow)		✔ (red) ✔ (blue) ✔ (yellow) ✔ (orange)			
Marker 4	✔ (yellow)	✔ (green) ✔ (yellow) ✔ (orange)	✔ (orange)			
Marker 5	✔ (yellow)	✔ (yellow) ✔ (orange)	✔ (yellow)			
Marker 6			✔ (orange)			
Marker 7						
Marker 8	✔ (red) ✔ (blue)					
Marker 9		✔ (orange)	✔ (orange)			
Marker 10	✔ (green) ✔ (yellow) ✔ (orange)		✔ (yellow)			
Marker 11			✔ (orange)			
Marker 12		✔ (orange)				
Marker 13	✔ (yellow) ✔ (orange)	✔ (yellow)				
Marker 14		✔ (orange)				
Marker 15	✔ (red) ✔ (blue) ✔ (yellow) ✔ (orange)	✔ (yellow)	✔ (yellow)			
Marker 16			✔ (orange)			
Marker 17	✔ (yellow) ✔ (orange)	✔ (yellow)				

Table S2: Summaries of predictors used to predict survival probability in primary biliary cholangitis patients.

**Predictor**	**Description**	**Type**	**Time-dependent Yes/No**
Bilirubin	Level of serum bilirubin	Continuous	Yes
Cholesterol	Level of serum cholesterol	Continuous	Yes
Albumin	Level of albumin	Continuous	Yes
Alkaline	Level of alkaline phosphatase	Continuous	Yes
SGOT	Level of aspartate aminotransferase	Continuous	Yes
Platelets	Platelet count	Continuous	Yes
Prothrombin	Prothrombin time	Continuous	Yes
Ascites	Presence of ascites (Yes/No)	Binary	Yes
Hepatomegaly	Presence of hepatomegaly (Yes/No)	Binary	Yes
Spiders	Blood vessel malformations in the skin (Yes/No)	Binary	Yes
Edema	Presence of edema (Yes/No)	Binary	Yes
Age	Age at enrollment	Continuous	No
Sex	/	Binary	No
Treatment	Drug treatment (D-penicillmain/Placebo)	Binary	No

Table S3: List of time-dependent variables used to predict the survival probability in the elderly

**Predictor**	**Description**	**Type**	**Time-dependent Yes/No**
Depression	Measured using the Center for Epidemiological Studies-Depression (CES-D) providing a score from 0 to 60, with high scores indicating most depressive symptoms	Continuous	Yes
Executive functioning	Measured using the Wechsler code test	Continuous	Yes
Cognition	Measured using the Mini-Mental State Examination (MMSE) providing a score from 0 to 30, with lower score indicating suspicion of dementia	Continuous	Yes
Verbal fluency	Measured using Isaac set Test, which evaluates the verbal fluency in 15 secondes by reapeated a list of specific words	Continuous	Yes
Dependency (IADL)	Measured using Instrumental Activities of Daily Living (IADL), also called Lawton scale, with multiple questions about how well you can live on your own	Continuous	Yes
Polymedication	Daily number of drugs taken by the patient	Continuous	Yes
Incontinence	Yes/No	Binary	Yes
Dyspnea	Yes/No	Binary	Yes
Living alone	Yes/No	Binary	Yes

Table S4: List of baseline variables used to predict the survival probability in the elderly

**Predictor**	**Description**	**Type**	**Time-dependent Yes/No**
Level of education	Scale from no education from higher education	5-level factor	No
Sex	Male/Female	Binary	No
Generation	Age at enrollment	Continuous	No
Hearing	Hearing self-assessment (Good/Medium/Bad)	3-level factor	No
Eyesight	Eyesight self-assessment (Good/Bad)	Binary	No
Diabete	Diabete history (Yes/No)	Binary	No
Stroke	Stroke history (Yes/No)	Binary	No
Dementia	Demantia history (Yes/No)	Binary	No
Fracture	Fracture history (Yes/No)	Binary	No
Family status	In couple/Single	Binary	No
City size	/	4-level factor	No
Owner	Owner/Tenant	Binary	No
Housing	Personal residence/Other	Binary	No
Satisfaction	Scale measuring whether the patient is satisfied with his life	Continuous	No
Health	Health self-assessment (Good/Medium/Bad)	3-level factor	No
Rural area	Yes/No	Binary	No
Cardiovascular	Cardiovascular history (Yes/No)	Binary	No
Dependency (ADL)	Measured using Activities of Daily Living (ADL), also called Katz scale, with multiple questions about the functional status of the patient	Binary	No
